# Association between acute kidney injury and prognoses of cardiac surgery patients: Analysis of the MIMIC-III database

**DOI:** 10.3389/fsurg.2022.1044937

**Published:** 2023-01-06

**Authors:** Chun Sun, Deqing Chen, Xin Jin, Guangtao Xu, Chenye Tang, Xiao Guo, Zhiling Tang, Yixin Bao, Fei Wang, Ruilin Shen

**Affiliations:** ^1^ Department of Surgery, Municipal Key-Innovative Discipline, the Second Affiliated Hospital of Jiaxing University, Jiaxing, China; ^2^ Forensic and Pathology Lab., Department of Pathology, Institute of Forensic Science, Jiaxing University, Jiaxing, China

**Keywords:** acute kidney injury, risk factor, prognosis, MIMIC-III database, cardiac surgery

## Abstract

**Background:**

Acute kidney injury (AKI) is the most common major complication of cardiac surgery field. The purpose of this study is to investigate the association between acute kidney injury and the prognoses of cardiac surgery patients in the Medical Information Mart for Intensive Care III (MIMIC-III) database.

**Methods:**

Clinical data were extracted from the MIMIC-III database. Adult (≥18 years) cardiac surgery patients in the database were enrolled. Multivariable logistic regression analyses were employed to assess the associations between acute kidney injury (AKI) comorbidity and 30-day mortality, 90-day mortality and hospital mortality. Different adjusting models were used to adjust for potential confounders.

**Results:**

A total of 6,002 patients were involved, among which 485 patients (8.08%) had comorbid AKI. Patients with AKI were at higher risks of prolonged ICU stay, hospital mortality, 90-day mortality (all *P* < 0.001), and 30-day mortality (*P* = 0.008). AKI was a risk factor for hospital mortality [Model 1, OR (95% CI) = 2.50 (1.45–4.33); Model 2, OR (95% CI) = 2.44 (1.48–4.02)], 30-day mortality [Model 1, OR (95% CI) = 1.84 (1.05–3.24); Model 2, OR (95% CI) = 1.96 (1.13–3.22)] and 90-day mortality [Model 1, OR (95% CI) = 2.05 (1.37–3.01); Model 2, OR (95% CI) = 2.76 (1.93–3.94)]. Higher hospital mortality, 30-day mortality and 90-day mortality was observed in higher KDIGO grade for cardiac surgery patients with AKI (all *P* < 0.05).

**Conclusion:**

Comorbid AKI increased the risk of hospital mortality, 30-day mortality, and 90-day mortality of cardiac surgery patients in the MIMIC-III database.

## Introduction

Acute kidney injury (AKI) is the most common major complication of cardiac surgery field (1). More than two million cardiac surgeries are performed throughout the world each year, and the incidence of cardiac surgery-associated AKI is between 5.0% and 42.0% (2, 3). Cardiac surgery-associated AKI (CSA-AKI) is independently associated with increased morbidity and mortality, and is the second most common cause of AKI in the intensive care unit (ICU) setting (post-sepsis) (4). Severe CSA-AKI is correlated with 3–8-fold higher perioperative mortality, as well as prolonged lengths of stay in the ICU and hospital (5). The risk of death associated with AKI remains high for 10 years after cardiac surgery regardless of other risk factors, even for patients with complete renal recovery (3). However, most of the current studies on AKI in patients undergoing cardiac surgery took all patients undergoing cardiac surgery as research objects, while few studies take patients undergoing cardiac surgery in intensive care unit as research objects. Cardiac surgery patients in the intensive care unit may be more affected by AKI because their condition is more severe.

The Medical Information Mart for Intensive Care (MIMIC)-III v 1.4 is an openly available US-based critical care database (6). The MIMIC-III is a large, integrated, deidentified, comprehensive clinical dataset. The database contains data on all patients treated in the ICUs of the Beth Israel Deaconess Medical Center (BIDMC) in Boston, MA, from June 1, 2001 to October 31, 2012. The database includes 380 laboratory measurements and hourly records, as well as basic information, laboratory results, imaging examinations, diagnoses, and other clinical information. The International Classification of Diseases, 9th revision (ICD-9) was used to describe precise diseases.

In the current study, we aimed to evaluate the association of AKI with hospital mortality, 30-day mortality, and 90-day mortality in patients from the MIMIC-III database who underwent on-pumped cardiac surgery.

## Methods

### Data source

We used a retrospective cohort study design from MIMIC-III database (6) and selected the clinical data of patients who stayed in the ICU of the BIDMC between 2001 and 2012. The institutional review boards of both the BIDMC and the Massachusetts Institute of Technology Affiliates approved the access to the database. No informed consent was required because all of the data were deidentified.

### Patient selection

Clinical data of eligible patients in the MIMIC-III database will be selected for entry into this study: (1) patients who underwent pump heart surgery; (2) patients with ages older than 18 years; and (3) patients with routine preoperative blood examinations within the first 24 h of admission.

Patients meeting criteria for AKI was according to the Kidney Disease: Improving Global Outcomes (KDIGO), which was also used in the MIMIC III database analysis reported previously (7). Briefly, AKI stages were defined by both serum creatinine and the volume of urine output during the first 48 h after ICU admission. Stage 1 was defined by increase in serum creatinine by ≥0.3 mg/dl (≥26.5 µmol/L) within 48 h after ICU admission or after cardiac surgery, or increase in serum creatinine of 1.5–1.9-fold of the baseline level, or urine output <0.5 ml/kg/h for 6–12 h. Stage 2 was defined by increase in serum creatinine of 2.0–2.9-fold of the baseline level, or urine output <0.5 ml/kg/h for ≥12 h. Stage 3 was defined by Increase in serum creatinine of 3.0-fold of the baseline level or increase in serum creatinine to ≥4.0 mg/dl (≥353.6 µmol/L), or urine output <0.3 ml/kg/h for ≥24 h or anuria for ≥12 h. Minimum of the serum creatinine values available within the 7 days before admission was used as the baseline serum creatinine. When the pre-admission serum creatinine was not available, the first serum creatinine measured at admission was used as the baseline serum creatinine.

### Data extraction

All of the data were obtained and extracted by using the Structured Query Language (SQL), and pgAdmin4 for PostgreSQL was used as the administrative platform. The extracted data mainly included demographics (age and sex), vital signs (diastolic blood pressure (DBP), heart rate (HR), respiratory rate (RR), systolic blood pressure (SBP), percutaneous oxygen saturation (SpO_2_), and temperature), comorbidities (AKI, cardiac arrhythmias, chronic pulmonary disease, congestive heart failure, hypertension, liver disease, peripheral vascular disorder, pulmonary circulation disorder, uncomplicated or complicated diabetes, and valvular disease), laboratory events (peripheral white blood cell count, platelet count, serum creatinine, serum glucose, serum potassium, and serum sodium), SAPS II and SOFA score, and coronary bypass artery grafting (CABG). When considering that the proportion of missing data for each variable was <1.5%, we directly omitted these data in further analyses.

### Outcome variables

The following outcome variables were extracted: 30-day and 90-day mortality (post-ICU-admission), hospital mortality, and ICU-stay length. Because the patient may have received more than one ICU hospitalization during a single hospitalization, the length of ICU stay is entirely determined by the first ICU hospitalization.

### Statistical analysis

The continuous variables are presented as the mean ± standard deviation or median (interquartile range), and were compared *via t*-test or Mann-Whitney *U* tests. The categorical data are presented as numbers with proportions and were analyzed *via* the *χ*^2^-test. Logistic regression with the uni-/multi-variate analyses was used for identifying independent prognostic factors of mortality (hospital, 30-day, and 90-day) after cardiac surgery. Two different models were designed for adjusting potential confounders. Model 1 was adjusted for cardiac arrhythmias, congestive heart failure, hypertension liver disease, SBP, and valvular disease. Moreover, Model 2 was adjusted for age, height, and weight. *P*-values of less than 0.05 were considered to indicate statistical significance. Further, we constructed the receiver operating characteristic (ROC) curves and calculated and analyzed the area under the curve (AUC), sensitivity, and specificity. All statistical analyses were performed using STATA, version 14.0 (StataCorp, College Station, TX).

## Results

### Baseline characteristics of the study population

In total, 6,002 patients who met the selection criteria participated in this study, among which 485 patients (8.08%) had AKI comorbidities. Table 1 briefly summarized the baseline characteristics of patients, including comorbidities, demographics, laboratory events, scores, and vital signs.

**Table 1:** Comparison of baseline characteristics between cardiac surgery patients with or without AKI.

	Without AKI (*n* = 5,517)	With AKI (*n* = 485)	*P* value
**Demographics**			
Age (years)	66.38 ± 12.26	66.69 ± 12.28	<0.001
Male, *n* (%)	3,795 (68.8%)	358 (73.8%)	0.022
Weight (kg)	81.8 (70.5–94.5)	81.1 (68–96.6)	0.973
Height (cm)	172.72 (162.56–177.8)	170.18 (162.56–177.8)	0.278
**Vital signs**			
HR, beats/minute	84.28 (78.49–90.63)	82.38 (77.36–87.38)	<0.001
SBP, mmHg	111.43 (105.80–118.07)	113.81 (107.41–121.14)	<0.001
DBP, mmHg	56.75 (52.81–61.16)	56.03 (50.86–60.00)	<0.001
RR, times/minute	16.86 (15.23–18.83)	16.89 (15.50–18.71)	0.398
Temperature, °C	36.84 (36.54–37.18)	36.72 (36.37–37.00)	<0.001
SpO_2_, %	98.13 (97.20–98.93)	98.34 (97.20–99.16)	0.124
**Laboratory events**			
WBC, 10^9^/L	12.1 (9.4–15.5)	11.8 (8.6–15.6)	0.100
Platelets, 10^9^/L	154 (122–196)	154 (121–201)	0.550
Glucose, mg/dl	126.9 (118.14–139.56)	124.33 (115.06–135.3)	0.018
Serum sodium, mmol/L	137 (135–138)	136 (134–138)	<0.001
Serum potassium, mmol/L	4.4 (3.9–5.1)	4.7 (4.2–5.3)	<0.001
Serum creatinine, mg/dl	0.8 (0.7–1)	1.4 (1.1–2.1)	<0.001
**Comorbidities**			
Congestive heart failure	1,316 (23.9%)	224 (46.2%)	<0.001
Cardiac arrhythmias	2,734 (49.6%)	280 (57.7%)	0.001
Valvular disease	1,190 (21.6%)	131 (27.0%)	0.006*
Pulmonary circulation disorder	388 (7.0%)	56 (11.5%)	<0.001
Peripheral vascular disorder	686 (12.4%)	103 (21.2%)	<0.001
Hypertension	3,782 (68.6%)	437 (90.1%)	<0.001
Chronic pulmonary	1,017 (18.4%)	121 (24.9%)	<0.001
Uncomplicated diabetes	1,414 (25.6%)	148 (30.5%)	0.019
Complicated diabetes	225 (4.1%)	115 (23.7%)	<0.001
Liver disease	140 (2.5%)	28 (5.7%)	<0.001
**Scores**			
SAPS II	32 (26–40)	41 (34–50)	<0.001
SOFA	4 (3–6)	6 (5–8)	<0.001
Surgical type			
CABG	3,825 (69.3%)	379 (78.1%)	<0.001

Values are presented as the mean ± standard deviation, median (interquartile range), or number of patients (%). AKI, acute kidney injury; CABG, coronary artery bypass grafting; DBP, diastolic blood pressure; HR, heart rate; RR, respiratory rate; SAPS II, Simplified Acute Physiology Score II; SBP, systolic blood pressure; SOFA, Sequential Organ Failure Assessment; SpO_2_, percutaneous oxygen saturation; WBC, white blood cell.

There are significant differences of baseline characteristics between the two groups. Cardiac surgery patients with AKI had a higher average age than patients without AKI (66.38 ± 12.26 vs. 66.69 ± 12.28, *P* < 0.001). More cardiac surgery patients with AKI were male, whereas the difference between the groups was little (68.8% vs. 73.8%, *P* = 0.022). Patients with AKI tended to have lower HR, DBP, temperature, glucose, and serum sodium, as well as higher SBP, RR, serum potassium, serum creatinine, SAPS II and SOFA scores, in addition to histories of cardiac arrhythmias, chronic pulmonary disease, congestive heart failure, hypertension, liver disease, peripheral vascular disorder, pulmonary circulation disorder, uncomplicated and complicated diabetes, and valvular disease (Table 1).

### Clinical outcomes of the study population

When compared with patients without AKI, patients with AKI were at higher risks of prolonged ICU stay, hospital mortality, 30-day mortality, and 90-day mortality (2.1 vs. 3.2 days, *P* < 0.001; 1.6% vs. 4.1%, *P* < 0.001; 1.8% vs. 3.5%, *P* = 0.008; 3.0% vs. 8.7%, *P* < 0.001, respectively) (Table 2).

**Table 2:** Outcomes of cardiac surgery patients with or without AKI.

	Without AKI (*n* = 5,517)	With AKI (*n* = 485)	*P* value
ICU stay, days	2.1 (1.2–3.7)	3.2 (1.9–5.9)	<0.001
Hospital mortality (%)	88 (1.6%)	20 (4.1%)	<0.001
30-day mortality (%)	98 (1.8%)	17 (3.5%)	0.008
90-day mortality (%)	165 (3.0%)	42 (8.7%)	<0.001

Values are presented as the median (interquartile range) or number of patients (%). AKI, acute kidney injury; ICU, intensive care unit.

A univariate-logistic-regression analysis was shown in Table 3, AKI, age, DBP, height, cardiac arrhythmias, congestive heart failure, hypertension, liver disease, peripheral vascular disorder, SBP, and valvular disease were associated with hospital mortality (all *P* < 0.01). Moreover, AKI, age, height, cardiac arrhythmias, congestive heart failure, hypertension, liver disease, peripheral vascular disorder, SBP, and valvular disease (all *P* < 0.01) were associated with 30-day mortality. Furthermore, AKI, age, DBP, cardiac arrhythmias, chronic pulmonary disease, complicated diabetes, congestive heart failure, height, hypertension, liver disease, peripheral vascular disorder, pulmonary circulation disorder, SBP, weight, and valvular disease (all *P* < 0.01) were associated with 90-day mortality.

**Table 3:** Univariate logistic regression analyses for prognosis in cardiac surgery patients.

Variable	Hospital mortality		30-day mortality		90-day mortality	
	OR (95% CI)	*P* value	OR (95% CI)	*P* value	OR (95% CI)	*P* value
AKI	2.65 (1.62–4.35)	<0.001	2.01 (1.19–3.39)	0.009	3.07 (2.16–4.37)	<0.001
Age	1.03 (1.02–1.05)	<0.001	1.02 (1.00–1.04)	0.018	1.04 (1.03–1.05)	<0.001
Weight	1.00 (0.99–1.01)	0.650	1.00 (0.99–1.01)	0.780	0.99 (0.98–1.00)	0.014
Height	0.96 (0.95–0.98)	<0.001	0.97 (0.95–0.99)	0.001	0.97 (0.96–0.98)	<0.001
Congestive heart failure	2.97 (2.03–4.34)	<0.001	2.72 (1.88–3.94)	<0.001	2.61 (1.97–3.45)	<0.001
Cardiac arrhythmias	2.09 (1.40–3.14)	<0.001	1.88 (1.28–2.77)	<0.001	2.38 (1.76–3.23)	<0.001
Valvular disease	1.72 (1.14–2.58)	0.009	1.99 (1.35–2.94)	<0.001	2.17 (1.62–2.98)	<0.001
Pulmonary circulation disorder	1.73 (0.96–3.12)	0.066	1.61 (0.90–2.90)	0.109	1.60 (1.02–2.49)	0.039
Peripheral vascular disorder	2.13 (1.36–3.34)	0.001	1.96 (1.26–3.06)	0.003	2.12 (1.52–2.95)	<0.001
Hypertension	0.42 (0.28–0.61)	<0.001	0.47 (0.32–0.68)	<0.001	0.56 (0.42–0.74)	<0.001
Chronic pulmonary	1.36 (0.87–2.13)	0.173	1.52 (1.00–2.32)	0.051	1.49 (1.09–2.06)	0.014
Uncomplicated diabetes	0.68 (0.42–1.10)	0.118	0.83 (0.53–1.29)	0.400	0.81 (0.58–1.12)	0.205
Complicated diabetes	1.72 (0.89–3.33)	0.107	1.43 (0.72–2.84)	0.314	1.72 (1.06–2.80)	0.028
Liver disease	12.84 (8.02–20.58)	<0.001	8.16 (4.90–13.58)	<0.001	8.66 (5.79–12.95)	<0.001
DBP	0.96 (0.93–0.99)	0.009	0.98 (0.95–1.01)	0.139	0.96 (0.94–0.98)	<0.001
SBP	0.95 (0.93–0.97)	<0.001	0.95 (0.93–0.97)	<0.001	0.98 (0.96–1.00)	0.015

AKI, acute kidney injury; CI, confidence interval; DBP, diastolic blood pressure; OR, odds ratio; SBP, systolic blood pressure.

**Figure 1:**
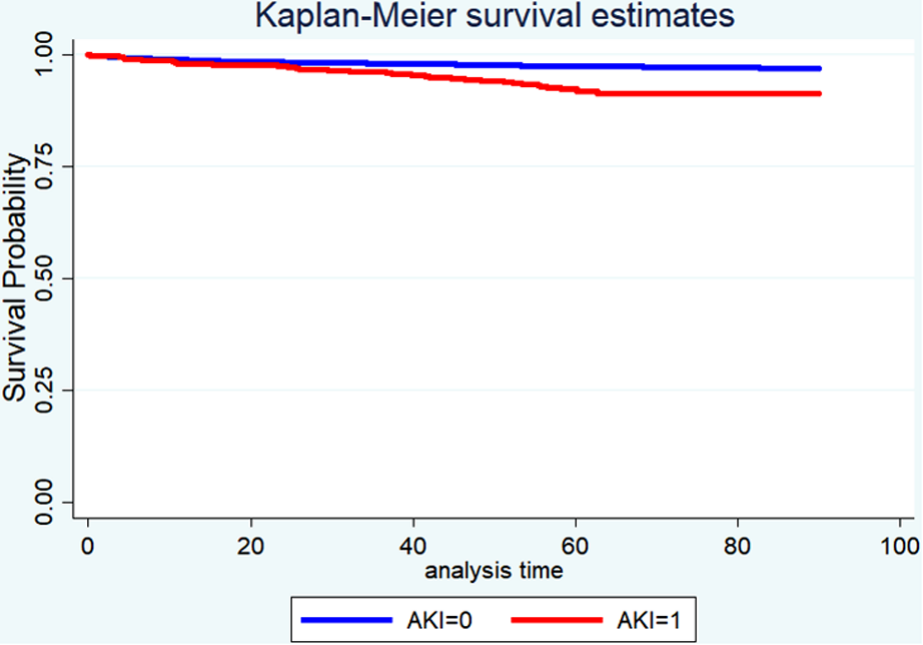
Kaplan-Meier survival analysis for 90-day overall survival.

Table 4 displayed the results of the multivariate analyses for Models 1 and 2. AKI was a risk factor for hospital mortality (Model 1: OR = 2.50, 95% CI: 1.45–4.33, *P* < 0.001; Model 2: OR = 2.44, 95% CI: 1.48–4.02, *P* < 0.001), 30-day mortality (Model 1: OR = 1.84, 95% CI: 1.05–3.24, *P* = 0.034; Model 2: OR = 1.96, 95% CI: 1.13–3.22, *P* = 0.016), and 90-day mortality (Model 1: OR = 2.05, 95% CI: 1.37–3.01, *P* < 0.001; Model 2: OR = 2.76, 95% CI: 1.93–3.94, *P* < 0.001). Furthermore, the Kaplan-Meier survival curves showed that cardiac surgery patients with AKI had a significantly lower 90-day survival rate compared with the patients without AKI (Figure 1).

**Table 4:** Multivariate logistic regression analyses for prognosis in cardiac surgery patients.

Model 1			Model 2		
Variable	OR (95% CI)	*P* value	Variable	OR (95% CI)	*P* value
**Hospital mortality**					
AKI	2.50 (1.45–4.33)	<0.001	AKI	2.44 (1.48–4.02)	<0.001
Congestive heart failure	2.05 (1.36–3.08)	0.001	Age	1.02 (1.01–1.04)	0.003
Cardiac arrhythmias	1.76 (1.15–2.69)	0.009	Height	0.97 (0.94–0.99)	<0.001
Valvular disease	1.11 (0.72–1.71)	0.628			
Liver disease	10.01 (6.10–16.42)	<0.001			
SBP	0.96 (0.94–0.98)	<0.001			
Hypertension	0.45 (0.30–0.67)	<0.001			
**30-day mortality**					
AKI	1.84 (1.05–3.24)	0.034	AKI	1.96 (1.13–3.22)	0.016
Congestive heart failure	1.96 (1.33–2.90)	0.001	Age	1.01 (1.00–1.03)	0.124
Cardiac arrhythmias	1.55 (1.04–2.32)	0.031	Height	0.97 (0.96–0.99)	0.003
Valvular disease	1.38 (0.93–2.01)	0.112			
Liver disease	6.24 (3.67–10.61)	<0.001			
SBP	0.96 (0.94–0.98)	<0.001			
Hypertension	0.54 (0.36–0.80)	0.002			
**90-day mortality**					
AKI	2.05 (1.37–3.01)	<0.001	AKI	2.76 (1.93–3.94)	<0.001
Congestive heart failure	1.48 (1.10–2.01)	0.011	Age	1.03 (1.02–1.04)	<0.001
Cardiac arrhythmias	1.82 (1.33–2.50)	<0.001	Height	0.98 (0.96–0.99)	0.001
Valvular disease	1.45 (1.07–2.00)	0.018			
Liver disease	5.28 (3.39–8.23)	<0.001			
SBP	0.99 (0.98–1.01)	0.455			
Hypertension	0.54 (0.40–0.73)	<0.001			

Model 1 was adjusted for cardiac arrhythmias, congestive heart failure, hypertension, liver disease, SBP, valvular disease. Model 2 was adjusted for age, height, weight. AKI, acute kidney injury; CI, confidence interval; OR, odds ratio; SBP, systolic blood pressure.

Table 5 showed that higher hospital mortality, 30-day mortality and 90-day mortality was observed in higher KDIGO grade for cardiac surgery patients with AKI (all *P* < 0.05).

**Table 5:** The relationship between KDIGO grade with mortality for cardiac surgery patients with AKI.

KDIGO grade	Hospital mortality			30-day mortality			90-day mortality		
	Survivors	Nonsurvivors	*P* value	Survivors	Nonsurvivors	*P* value	Survivors	Nonsurvivors	*P* value
1	168 (34.64%)	2 (0.04%)	**0** **.** **04**	170 (35.05%)	2 (0.04%)	**0** **.** **023**	169 (34.85%)	4 (0.82%)	**0** **.** **001**
2	154 (31.75%)	5 (1.03%)		161 (33.20%)	4 (0.82%)		142 (29.28%)	6 (1.24%)	
3	143 (29.48%)	13 (2.68%)		137 (28.25%)	11 (2.27%)		132 (27.22%)	32 (6.60%)	

Values are presented as the number of patients (%). AKI, acute kidney injury; KDIGO, kidney disease: improving global outcomes.

### Predictive ability of creatinine for AKI

**Figure 2:**
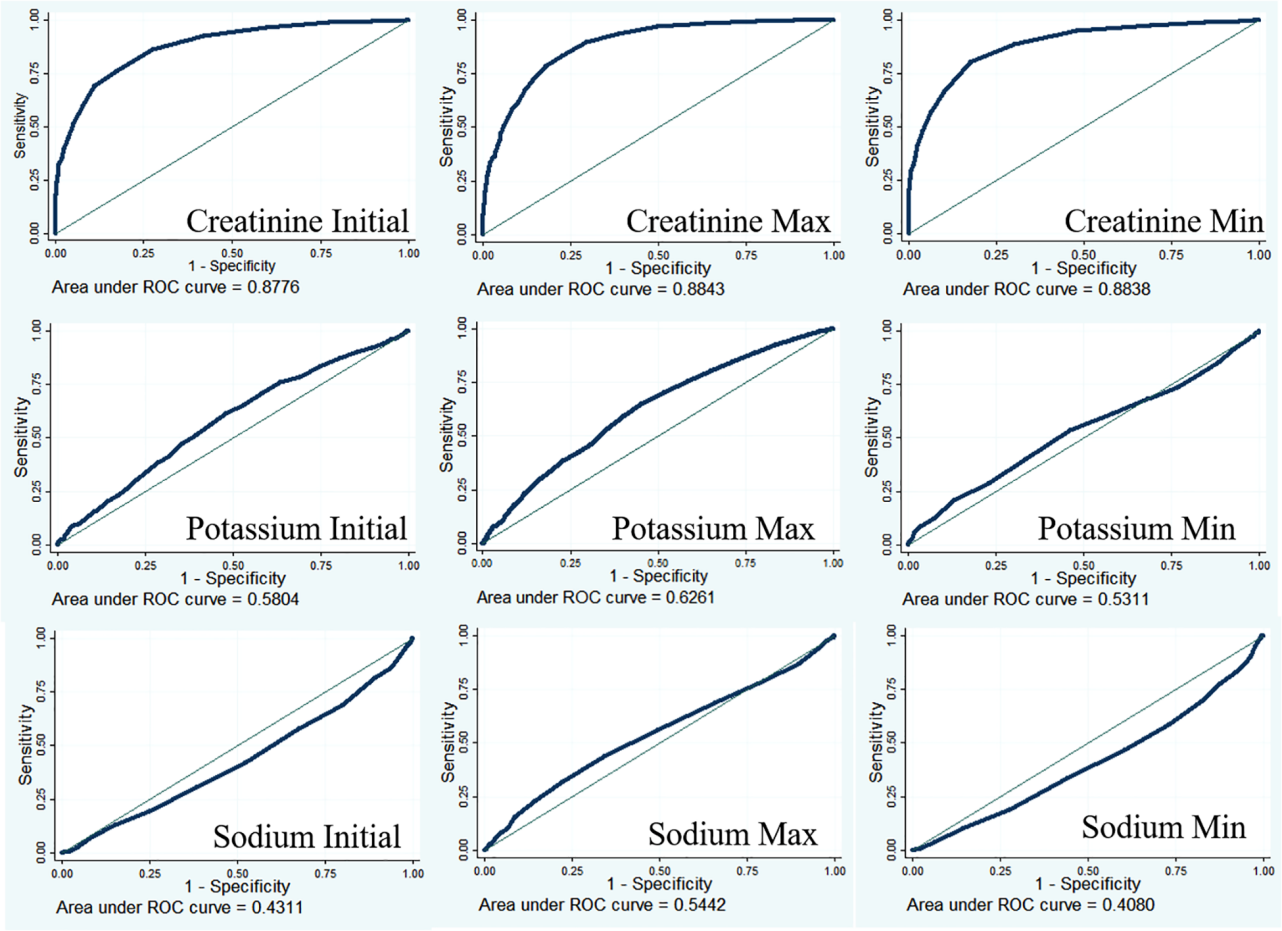
Receiver operating characteristic curves for creatinine, potassium, and sodium in AKI.

Creatinine is an important diagnostic indicator for AKI (8). The diagnostic value of creatinine was examined using receiver operating characteristic curves (Figure 2). The results showed that the diagnostic performance of creatinine was moderately good (AUC for creatinine initial = 0.8776; AUC for creatinine max = 0.8843; AUC for creatinine min = 0.8838), and a low AUC was found in potassium (AUC for potassium initial = 0.5804; AUC for potassium max = 0.6261; AUC for potassium min = 0.5311) and sodium (AUC for sodium initial = 0.4311; AUC for sodium max = 0.5442; AUC for sodium min = 0.4080) detection.

## Discussion

In the present study, we found that the incidence of AKI in cardiac surgery patients was 8.08%. Patients with AKI had a higher average age than patients without AKI, and more patients with AKI were male. When compared with patients without AKI, patients with AKI were at higher risks of prolonged ICU stay, hospital mortality, 30-day mortality, and 90-day mortality. Higher hospital mortality, 30-day mortality and 90-day mortality was observed in higher KDIGO grade for cardiac surgery patients with AKI. In the multivariate analysis, AKI was a risk factor for hospital mortality, 30-day mortality, and 90-day mortality. The diagnostic performance of creatinine was moderately good for AKI.

According to the literature, the mortality rate of all patients undergoing cardiac surgery is as high as 8%, and that of patients with postoperative complications of AKI can be as high as 60% or more (9). Patients undergoing cardiac surgery will increase mortality from 0.4–4.4% to 1.3–22.3% once complicated AKI; when these same patients need clinical hemodialysis, the mortality rate rises from 25% to 88.9%, thus indicating that severe postoperative complications AKI an independent risk factor for mortality, resulting in an eightfold increase in the risk of death (10, 11). Therefore, the postoperative AKI of cardiac surgery is closely related to other complications, ICU hospital stay, and worse quality of life. These also indicate an increase in the early and late mortality rates of such populations and in health care financial expenditures (5, 12, 13). Our data is consistent with previous studies. In the present study, patients with AKI had longer ICU stays (2.1 vs. 3.2 days), as well as higher hospital mortality (1.6% vs. 4.1%), 30-day mortality (1.8% vs. 3.5%), and 90-day mortality (3.0% vs. 8.7%) than patients without AKI. Moreover, higher hospital mortality, 30-day mortality and 90-day mortality was observed in higher KDIGO grade for cardiac surgery patients with AKI (all *P* < 0.05, Table 5). The development of AKI in cardiac surgery might be connected with several major injury pathways, including hypoperfusion, ischemia-reperfusion injury, inflammation, oxidative stress, nephrotoxins, neurohumoral activation and mechanical factor, all of which could occur preoperatively, intraoperatively and postoperatively. Although these factors have been associated with AKI in cardiac surgery, the current evidence was associative rather than causal (1). Furthermore, our understanding of the pathophysiology of AKI and cardiac surgery remained rudimentary due to the huge logistic challenges and costs, as well as the lacking of animal models of cardiac surgery. Therefore, the lack of understanding of the pathogenesis of AKI greatly limits the development of prevention and management measures for AKI in cardiac surgery. So far, AKI is still one of the important factors affecting the mortality and ICU stay in cardiac surgery patients.

Common preoperative risk factors for the development of AKI after cardiac surgery include age, obesity, the female sex, the presence of multiple comorbidities (for example, chronic obstructive pulmonary disease, congestive heart failure, diabetes mellitus, hypertension, hypercholesterolaemia, left ventricular ejection fraction of <35%, pre-existing chronic kidney disease, and previous cardiac surgery), and obesity (14–16). In our investigation, patients with AKI tended to have lower HR, DBP, temperature, glucose, and serum sodium, as well as higher SBP, RR, creatinine and potassium of serum, SAPS II and SOFA score, in addition to a history of cardiac arrhythmias, chronic pulmonary disease, congestive heart failure, hypertension, liver disease, peripheral vascular disorder, pulmonary circulation disorder, uncomplicated and complicated diabetes, and valvular disease. Hence, it is important to identify risk factors for concurrent AKI in patients undergoing cardiac surgery, which can facilitate clinical prognostic assessment and develop new and more effective clinical strategies to prevent and reduce this complication, thereby reducing associated morbidity and mortality.

Serum creatinine is an important diagnostic indicator for AKI (8). Under normal physiological conditions, serum levels of creatinine increase by 0.1–0.2 mg/dl after cardiac surgery. When creatinine levels increase by more than 0.3 mg/dl, within 2 days, patients are considered stage 1 AKI according to KDIGO criteria (17). In the present study, serum creatinine showed a moderately good diagnostic performance for AKI (AUC for creatinine initial = 0.8776; AUC for creatinine max = 0.8843; AUC for creatinine min = 0.8838). In addition to creatinine, a series of new biomarkers for the early diagnosis and prognostication of AIK have been proposed, such as NGAL (18–20), cystatin C (19), and IL-18 (21, 22). However, these biomarkers showed poor predictive performances in these patients, with a heterogeneous timing of the onset of injury (23).

## Limitation

Our investigation has some limitations. Firstly, the definition of AKI changed in 2012, while the MIMIC-III database only contains data on critically ill patients admitted between 2001 and 2012. Although we tried to identify the AKI patients according to KDIGO, our cohort may not fully comply with the newly defined AKI. What's more, transient and persistent AKI, as well as severity degree of AKI were not been obtained. Secondly, this was a single center study. The results need to be verified by multi-center trials. Moreover, the disease definition of the MIMIC-III database was based on the ICD-9-CM code, which may lead to some important information lacking in the database. Then, it was a retrospective design and it is difficult to completely eliminate residual confounding. Due to the retrospective nature the temporal relationship is frequently difficult to assess. Furthermore, the findings in this study may not be generalizable to the up-to-date ICD-10 codes or other data sources or hospitals as MIMIC-III data comes from a single hospital system. Moreover, the present results could only show the correlation between AKI and mortality, but not the causal relationship. To solve this problem, causal inference could be used in the future as the previous study (24).

## Conclusion

In summary, we found that AKI can lead to prolonged ICU stay, hospital mortality, 30-day mortality, and 90-day mortality. AKI was a risk factor for hospital mortality, 30-day mortality, and 90-day mortality. The diagnostic performance of creatinine was moderately good for AKI.

## Data Availability

The original contributions presented in the study are included in the article/Supplementary Material, further inquiries can be directed to the corresponding author.
